# Correlation Between Dental Health and Aesthetic Components of Malocclusion in Junior High and High School Students: An Epidemiological Study Using Item Response Theory

**DOI:** 10.3390/jcm14134802

**Published:** 2025-07-07

**Authors:** Hiromi Sato, Yudai Shimpo, Toshiko Sekiya, Haruna Rikitake, Minami Seki, Satoshi Wada, Yoshiaki Nomura, Hiroshi Tomonari

**Affiliations:** 1 Department of Orthodontics, School of Dental Medicine, Tsurumi University, Yokohama 230-8501, Japan; sato-h1112@outlook.jp (H.S.); sekiya-t@tsurumi-u.ac.jp (T.S.); shanty0330@gmail.com (H.R.); ssaw0209@icloud.com (M.S.); tomonari-h@tsurumi-u.ac.jp (H.T.); 2 Department of Oral and Maxillofacial Surgery, Kanazawa Medical University, Kanazawa 920-0293, Ishikawa, Japan; wada-s@kanazawa-med.ac.jp; 3 Institute of Photochemistry and Photocatalyst, University of Shanghai for Science and Technology, Shanghai 20093, China; nomura-y@sirius.ocn.ne.jp

**Keywords:** epidemiological survey, item response theory, index of orthodontic treatment need, dental health component, aesthetic component

## Abstract

**Background:** The Index of Orthodontic Treatment Need (IOTN) is widely used to assess the need for orthodontic treatment. IOTN consists of the Dental Health Component (DHC) and the Aesthetic Component (AC), evaluating malocclusion morphologically and aesthetically, respectively. However, the discriminatory power of individual DHC items and their relationship with AC grades remain unclear. **Objective:** This study aimed to evaluate the effectiveness of individual DHC items in school dental examinations and investigate their contribution to AC grades among junior high and high school students. **Methods:** A total of 726 students (443 males, 283 females; aged 12–18 years) from Tsurumi University Junior and Senior High School, excluding 168 students undergoing or having completed orthodontic treatment, were included. Nine calibrated orthodontists assessed DHC and AC using IOTN during standardized school examinations. The discriminatory power and information precision of DHC items were evaluated by Item Response Theory (IRT) analysis using three-, two-, or one-parameter logistic models depending on convergence. Correspondence analysis visualized the correlation between DHC and AC grades. Simple linear regression analyzed the contribution of each DHC item to AC grades. **Results:** Orthodontic treatment need was identified in 21.1% of students. Females showed a higher rate of treatment need than males. Correspondence analysis suggested that aesthetic evaluations were more lenient than morphological evaluations. IRT and regression analysis revealed that crowding (4.d), increased overjet (2.a), and increased overbite (2.f) demonstrated high discriminatory power and significant contributions to AC grades. **Conclusions:** Among the DHC items, crowding, increased overjet, and increased overbite had higher discriminatory power for malocclusion and contributed more significantly to AC evaluations compared to other items.

## 1. Introduction

Malocclusion not only affects oral functions, quality of life, mental health, and social well-being, but it is also recognized as a risk factor for major oral diseases such as dental caries and periodontal disease [1,2,3]. Consequently, orthodontic treatment is often recommended for individuals with malocclusion to improve oral function, aesthetics, and quality of life. It has been reported that approximately 30% of the global population requires orthodontic treatment [4,5].

Several indices have been developed to evaluate the need for orthodontic treatment, including the Index of Orthodontic Treatment Need (IOTN) [6], Dental Aesthetic Index (DAI) [7], Discrepancy Index (DI) [8], Treatment Priority Index [9], Occlusion Feature Index [10], and the American Board of Orthodontics Objective Grading System [11]. Among these, only IOTN and DAI assess malocclusion from both morphological and aesthetic perspectives; the others focus solely on morphological evaluation [12].

While DAI has been widely used in epidemiological studies of malocclusion [7,13], it requires detailed measurements of multiple aesthetic-related factors, making it labor-intensive and less suitable for large-scale surveys [14]. In contrast, IOTN evaluates only the most severe features of malocclusion, offering a simpler and more time-efficient assessment, especially suitable for epidemiological studies and healthcare planning [15]. IOTN is currently used by the National Health Service (NHS) in the United Kingdom to determine eligibility for orthodontic treatment [4].

IOTN consists of two components: the Dental Health Component (DHC), which assesses morphological aspects of malocclusion, and the Aesthetic Component (AC), which evaluates aesthetic concerns. However, DHC evaluates only the most severe morphological features of malocclusion [16], potentially overlooking other relevant features [17]. Although indices such as the Index of Orthodontic Treatment Need (IOTN) are widely used to assess malocclusion severity and the need for orthodontic treatment, it is important to note that a functional malocclusion does not always compromise facial esthetics. In many cases, patients may achieve an esthetically pleasing smile without complete correction of malocclusion. According to Chrapla et al. (2022) [18], the concept of the golden proportion and the relative symmetry of dental and facial components play a significant role in the perception of smile esthetics. Their study highlights that aesthetic satisfaction is not always contingent on ideal occlusion but can be significantly influenced by harmonious tooth proportions and facial balance. Therefore, orthodontic evaluation should include both functional and esthetic considerations, acknowledging that subjective perceptions of beauty may vary and may not always align with objective occlusal indicators. Furthermore, the discriminatory power and validity of individual DHC items in evaluating malocclusion severity remain unclear.

Item Response Theory (IRT) is a psychometric model widely used to evaluate the quality and discriminatory power of test items in large-scale examinations [19,20]. Applying IRT to DHC items may provide insights into the discriminatory power and precision of each item, enabling a more nuanced understanding of malocclusion assessment.

Although DHC and AC are measured independently, both are critical factors influencing the perceived need for orthodontic treatment. Clarifying the relationship between individual DHC items and AC grades is essential for a comprehensive evaluation of malocclusion severity and treatment need. However, little is known about the detailed correlation between DHC items and AC, as well as the contribution of each DHC item to AC grades [21,22].

Therefore, the aim of this study was to evaluate the effectiveness of individual DHC items using IRT and to investigate their contribution to AC grades in a population of junior high and high school students undergoing school-based dental examinations.

## 2. Materials and Methods

### 2.1. Study Design and Population

This cross-sectional study was conducted among students at Tsurumi University Junior and Senior High School who underwent a school dental examination on 15 and 16 April 2021. A total of 894 students (aged 12–18 years) were invited to participate. Of these, 726 students (443 males and 283 females) provided written informed consent and completed the examination, resulting in a participation rate of 81.2%. All participants were of Japanese ethnicity (Mongoloid).

Students who were currently undergoing orthodontic treatment (*n* = 122) or had completed orthodontic treatment in the past (*n* = 46) were excluded from the analysis. Therefore, 168 students (18.8%) were excluded, and 726 students were included in the final analysis. The clinical evaluations were conducted by nine licensed orthodontists with standardized calibration training. This study was conducted in accordance with the Declaration of Helsinki and approved by the Institutional Review Board of the School of Dental Medicine, Tsurumi University (Approval No. 1752; approved on 25 March 2020).

### 2.2. Clinical Examination

Orthodontic treatment need was assessed using the Index of Orthodontic Treatment Need (IOTN), which includes the Dental Health Component (DHC) and Aesthetic Component (AC). Nine board-certified orthodontists from the Department of Orthodontics, Tsurumi University School of Dental Medicine, conducted the oral examinations. All examiners underwent a calibration session prior to data collection to standardize assessment procedures. Inter-examiner reliability was evaluated using the kappa statistic, resulting in a value of 0.26, indicating fair agreement.

Examinations were performed in classrooms under standardized conditions, using portable LED dental lights, dental mirrors, and WHO periodontal probes (YDM, Tokyo, Japan). Students were examined while seated upright, following the same protocol regardless of school grade or gender. Oral evaluations included assessment of anterior, posterior, and lingual crossbites; degree of crowding in both arches; presence of cleft lip and/or palate; clinical signs suggestive of congenital absence of permanent teeth (e.g., prolonged retention of primary teeth or lack of eruption space for permanent successors); overjet; and overbite. Overjet and overbite were measured on the left maxillary central incisor using a WHO probe. The presence or absence of orthodontic treatment history was confirmed via individual interviews prior to the examination.

### 2.3. Outcome Assessment

DHC grades were determined according to the IOTN criteria (Brook and Shaw, 1989) [1] based on the most severe feature of malocclusion present in each student. DHC grades were recorded on a 5-point scale from grade 1 (minor malocclusion) to grade 5 (severe malocclusion). AC grades were assessed by comparing students’ anterior dentition to a set of 10 standardized monochrome photographs representing increasing levels of dental aesthetic impairment, using the original English version of the IOTN photographs. Each student was evaluated by one of the nine orthodontists. The assigned examiner independently selected the photograph from the AC reference chart that most closely resembled the student’s anterior dentition.

Orthodontic treatment need was categorized according to Richmond’s criteria [23], as follows:

Treatment required: DHC grade 4 or 5, or AC grade 8–10.

Borderline need: DHC grade 3, or AC grade 5–7.

No treatment needed: DHC grade 1 or 2, or AC grade 1–4.

A visual summary of this classification system is provided in Supplemental Figure S1 (Flowchart of IOTN Classification and Orthodontic Treatment Need Categories).

### 2.4. Statistical Analysis

Descriptive statistics were calculated for DHC and AC grades stratified by school grade and gender. Associations between categorical variables were evaluated using Fisher’s exact test. Item Response Theory (IRT) analyses were performed for each DHC grade to estimate discrimination, difficulty, and guessing parameters. A three-parameter logistic model was initially applied; if convergence failed, a two-parameter model was used, and if convergence still failed, a one-parameter (Rasch) model was applied. Item characteristic curves and item information curves were generated.

Correspondence analysis was conducted to explore associations between DHC and AC grades. Logistic regression models were constructed to assess the discriminatory power of each Aesthetic Component (AC) grade cutoff in identifying high orthodontic treatment need (DHC grade 4 or 5), as an alternative to receiver operating characteristic (ROC) analysis. While conceptually related to ROC curves, these models report regression coefficients instead of AUC values [24,25]. Simple linear regression analyses were performed to estimate the contribution of each DHC item to AC grades.

All statistical analyses, except for IRT analyses, were performed using SPSS Statistics version 27 (IBM, Tokyo, Japan). IRT analyses were performed using R version 4.0.3 with the “ltm” and “irtoys” packages. No missing data were identified for any of the variables assessed. The complete individual-level dataset used for analysis is provided in Supplemental File S1. The R code used for IRT analysis is available in Supplemental File S2.

### 2.5. Ethical Consideration

The study protocol was approved by the Research Ethics Committee of Tsurumi University School of Dental Medicine (Approval No. 1752; approved on 25 March 2020). This study was conducted in accordance with the principles of the Declaration of Helsinki. Written informed consent was obtained from all participants and their legal guardians prior to participation.

## 3. Results

### 3.1. Descriptive Statistics

#### 3.1.1. Sample Characteristics and Treatment Need Classification

Of the 894 students initially enrolled, 168 students (18.8%) were excluded because they were undergoing or had completed orthodontic treatment. The final sample consisted of 726 students (443 males and 283 females). According to the Index of Orthodontic Treatment Need (IOTN) Dental Health Component (DHC), 153 students (21.1%) were classified as requiring orthodontic treatment (DHC grade 4 or 5), 200 students (27.5%) as borderline (DHC grade 3), and 374 students (51.5%) as having no treatment need (DHC grade 1 or 2) (Table 1A).

#### 3.1.2. Inter-Examiner Reliability

Inter-examiner reliability among the nine orthodontists, evaluated using the kappa statistic, was 0.26, indicating fair agreement.

#### 3.1.3. Distribution of DHC and AC Grades by Grade and Gender

A breakdown of DHC grades by school grade and gender is presented in Table 1A. The prevalence of orthodontic treatment need was higher among females compared to males across both junior high and high school levels. In particular, treatment need was greater among second- and third-year junior high school girls and among second- and third-year high school students of both genders.

The distribution of Aesthetic Component (AC) grades is summarized in Table 1B. The proportion of students with poor aesthetic grades (AC grades 8–10) was relatively low across all grades, suggesting that aesthetic dissatisfaction was less prevalent than morphological malocclusion.

#### 3.1.4. Association Between DHC and AC Grades

**Figure 1:**
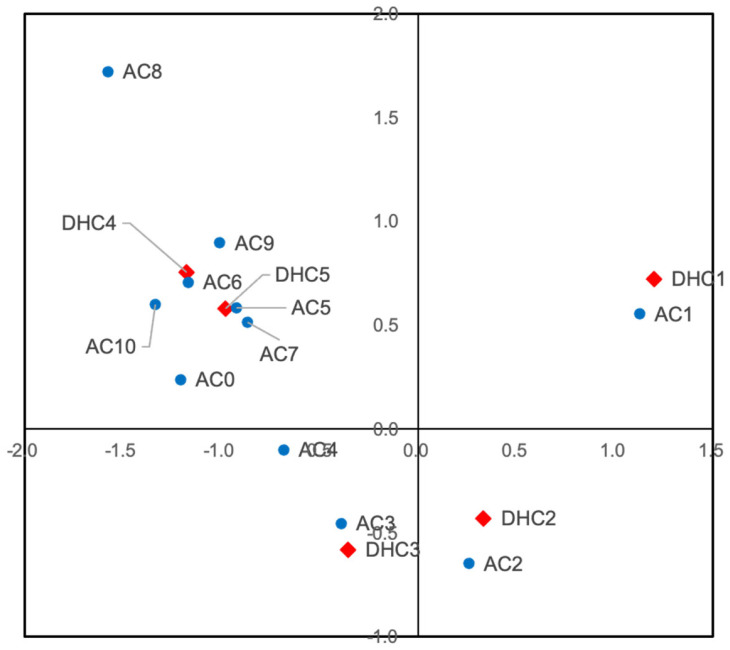
Correspondence analysis plot showing the association between Dental Health Component (DHC) grades and Aesthetic Component (AC) grades of the Index of Orthodontic Treatment Need (IOTN). Red diamonds represent DHC grades, and blue circles represent AC grades. The spatial proximity between markers reflects the strength of association; for example, DHC grades 4–5 cluster with AC grades 5–10, indicating alignment at higher severity levels, while a shift toward lower AC grades suggests underestimation of treatment need based on aesthetic assessment.

A cross-tabulation of DHC and AC grades is presented in Table 2. A significant association was observed between DHC and AC grades (Fisher’s exact test, *p* < 0.001). Correspondence analysis (Figure 1) revealed a clear clustering pattern: DHC grade 1 was closely associated with AC grade 1, DHC grade 2 with AC grade 2, DHC grade 3 with AC grades 3–4, and DHC grades 4–5 with AC grades 5–10. While these results indicate alignment across severity levels, a consistent shift pattern was also observed, in which AC grades tended to be slightly lower than the corresponding DHC grades. This suggests that subjective aesthetic evaluations may underestimate the level of orthodontic treatment needed compared to morphological assessments based on DHC.

### 3.2. Item Response Theory (IRT) Analysis of DHC Items

**Figure 2:**
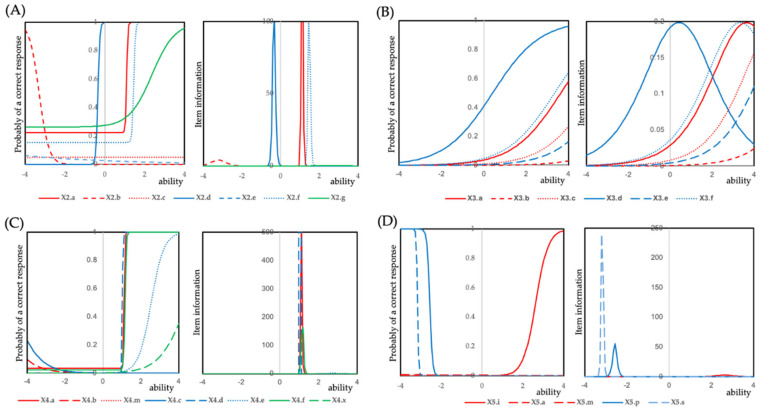
Item response curves and item information curves for items in each grade of the Dental Health Component (DHC). (**A**) Grade 2, (**B**) Grade 3, (**C**) Grade 4, and (**D**) Grade 5. A three-parameter logistic model was applied to Grades 2 and 4, a one-parameter logistic model (Rasch model) to Grade 3, and a two-parameter logistic model to Grade 5. For Grade 3 and Grade 5, convergence could not be achieved using the two-parameter and three-parameter logistic models, respectively. The applied models are summarized in Table 3.

IRT analysis was performed for each DHC grade to evaluate the discriminatory power and information precision of individual DHC items (Figure 2 and Table 3 and Table S1). The number of students who were positive for each DHC item is summarized in Table S1, providing the distribution of positive findings by school grade and gender and illustrating the prevalence of each morphological feature across the sample. The discrimination, difficulty, and guessing parameters estimated for each DHC item are summarized in Table 3. Table 3 shows that items such as crowding (4.d), increased overjet (4.a), and increased overbite (4.f) exhibited the highest discrimination parameters across grades, indicating their strong ability to differentiate between students with and without orthodontic treatment needs. In contrast, items such as reverse overjet (4.b, 5.m) and molar relationship (2.g) showed lower discrimination values, reflecting their limited contribution to distinguishing treatment need based solely on morphological severity.

For DHC grade 2, items 2.a (increased overjet), 2.d (crowding), and 2.f (increased overbite) demonstrated high discrimination (36.15, 21.13, and 25.00, respectively), while item 2.g (molar relationship) showed moderate discrimination (1.74). For DHC grade 4, items 4.a (increased overjet), 4.d (crowding), and 4.f (increased overbite) showed high discrimination (48.65, 243.28, and 29.92, respectively). For DHC grade 5, item 5.i (impeded eruption of teeth) exhibited moderate discrimination (3.28).

Item characteristic curves and item information curves for each DHC grade are illustrated in Figure 2. These results indicate that increased overjet, crowding, and increased overbite were consistently strong discriminators across different DHC grades. Conversely, items such as reverse overjet (4.b, 5.m) showed low discrimination in IRT analysis despite their functional relevance, suggesting that morphological severity alone may not capture their clinical impact.

### 3.3. Regression Analysis

Cutoff-specific logistic regression analysis showed that all Aesthetic Component (AC) grade thresholds were statistically significant predictors of orthodontic treatment need (*p* < 0.05) (Table S2A), indicating that AC grades could effectively distinguish students with high treatment need as defined by DHC grades 4 or 5. Although conceptually related to ROC analysis, this approach reports regression coefficients rather than AUC values, aligning with standard methodologies for logistic regression modeling [23,24].

Simple linear regression analysis identified significant predictors of AC grades (Table S2B). For DHC grade 2, significant predictors included Increased overjet (2.a: *p* = 0.003), Anterior or posterior crossbite (2.c: *p* = 0.001), Crowding (2.d: *p* < 0.001), Open bite (2.e: *p* < 0.001), Increased overbite (2.f: *p* = 0.003), and Molar relationship (2.g: *p* < 0.001). For DHC grade 3, item 3.d (Crowding) was a significant predictor (*p* < 0.001). For DHC grade 4, items 4.b (reverse overjet without reported functional difficulty; *p* = 0.001) and 4.d (Crowding; *p* < 0.001) were significant. For DHC grade 5, item 5.m (reverse overjet with reported masticatory or speech difficulty) was significant (*p* = 0.021).

Interestingly, some items such as reverse overjet (4.b, 5.m) were statistically significant in regression analysis despite low discrimination in IRT, implying that functional or perceptual factors might influence their impact on aesthetic evaluation. This suggests that features with functional impairment may affect subjective perception more than their morphological severity alone would predict.

## 4. Discussion

This study applied Item Response Theory (IRT) to evaluate the discriminatory power of individual items in the Dental Health Component (DHC) of the Index of Orthodontic Treatment Need (IOTN) in a school-based population. Our findings provide important insights into the differential contribution of morphological features to orthodontic treatment need and aesthetic perception.

The overall prevalence of orthodontic treatment need in this study was 21.1%, slightly lower than previous reports from Japan, which ranged from 32.5% to 35.5% [26]. Treatment need was higher among females, particularly in later school years, possibly reflecting gender differences in growth patterns or aesthetic awareness [27,28]. Although the number of males in DHC grade 5 was slightly higher than females, the overall prevalence of treatment need (DHC grades 4 and 5 combined) was higher in females. This may reflect a greater proportion of females classified in DHC grade 4 and suggests that while severe malocclusion (grade 5) may be more common among males, moderate-to-severe cases (grade 4) are more prevalent among females in this population.

IRT analysis demonstrated that crowding, increased overjet, and increased overbite consistently showed high discrimination and information across multiple DHC grades, supporting their role as key morphological indicators of malocclusion severity [4,5,26]. Our findings emphasize the clinical relevance of crowding, increased overjet, and increased overbite as key morphological features influencing both objective and subjective perceptions of malocclusion severity.

The correspondence analysis revealed that although DHC and AC grades were generally aligned, aesthetic assessments tended to underestimate treatment need compared to morphological evaluations [12,16]. These findings suggest that aesthetic assessments alone may not adequately capture the clinical severity of malocclusion, highlighting the importance of integrating both components in treatment planning. Additionally, the use of photographic comparison in AC grading is inherently subjective and may vary among examiners. Although all examiners underwent calibration, future studies may benefit from the use of standardized photographic documentation and consensus-based scoring to improve reliability.

Although our study did not directly evaluate patient preferences, previous studies have suggested that patients and their guardians may prioritize aesthetic improvement, even when morphological issues are mild, whereas severe morphological issues with low aesthetic impact may be underestimated without professional evaluation [16,20]. Several studies have shown that malocclusion can negatively influence an individual’s oral health-related quality of life (OHRQoL), especially in adolescents. Barrera-Chaparro et al. (2023) reported that beyond functional limitations, malocclusion may affect psychological well-being, self-esteem, and social interactions [29]. Such psychosocial impacts often play a role in a patient’s motivation to seek orthodontic treatment. Accordingly, the assessment of orthodontic treatment need should not only be based on objective indices but also consider the patient’s subjective perception and quality of life. This perspective supports the integration of both clinical criteria and patient-reported outcomes in treatment planning and screening strategies.

Interestingly, regression analysis identified significant associations for items such as reverse overjet (4.b, 5.m), despite their low discrimination in IRT. This discrepancy may reflect the influence of functional problems (e.g., speech and mastication) or perceptual factors that are not fully captured by morphological criteria [17,22]. The presence of functional impairment, such as masticatory difficulties or speech disturbances, may elevate the perceived need for treatment even when morphological severity appears moderate [10,17]. This observation aligns with earlier reports that patient-perceived treatment need is influenced by both functional and aesthetic concerns [7,12].

Furthermore, the observed underestimation of treatment need by AC grades in comparison to DHC grades suggests that aesthetic perception may be culturally or individually influenced, as previously reported in international comparisons [21,22]. Cultural differences in dental esthetics, peer influence, and media exposure have been shown to affect adolescents’ self-perception of malocclusion [19,21,28].

Interestingly, we observed that several students classified in DHC grades 4 and 5 had relatively low AC grades. This discrepancy may be attributed to the nature of the AC, which primarily assesses anterior aesthetic impairments based on frontal views. Morphological issues such as deep bites, crossbites, or posterior discrepancies may be severe functionally but remain less noticeable aesthetically.

These findings highlight the limitation of relying solely on AC for screening and emphasize the importance of combining morphological and aesthetic assessments. While our findings showed a general alignment between DHC and AC grades, we also observed cases where AC underestimated treatment need identified by DHC, particularly in students with deep bite or posterior malocclusion. Therefore, although AC may serve as a useful initial screening tool due to its simplicity, it should be complemented by DHC or clinical examination to ensure that functionally severe cases are not overlooked.

Our findings have practical implications for school-based dental screening programs. Reliance solely on aesthetic evaluation may fail to identify students requiring orthodontic intervention for functional or morphological reasons. To ensure an unbiased assessment of malocclusion severity and treatment need, we excluded participants who were currently undergoing orthodontic treatment or had completed treatment in the past. Including such individuals could have confounded the results by altering their morphological features, making it difficult to interpret the relationship between IOTN components and natural malocclusion patterns. This approach allowed for a clearer evaluation of untreated malocclusion in a general school population. Incorporating objective indices such as IOTN-DHC along with AC can improve screening accuracy and ensure that students with clinically significant malocclusion are appropriately referred [4,9].

Limitations of this study include its single-center design, limiting generalizability to broader populations. Additionally, we did not adjust for potential confounding factors such as oral habits, socioeconomic status, or previous dental interventions, which may influence both morphological and aesthetic assessments [19,20]. Further research incorporating larger, multicenter samples and adjustment for potential confounders is warranted to validate these findings and inform public health strategies. In addition, longitudinal studies are needed to explore how malocclusion severity and treatment needs evolve over time, particularly during adolescent growth phases [27,28]. Another limitation is that the assessment of congenitally missing permanent teeth was based solely on clinical signs, such as retained primary teeth or absence of eruption space. Although these signs may suggest agenesis, we acknowledge that radiographic confirmation is necessary to establish a definitive diagnosis. The absence of imaging may have led to misclassification, particularly in younger participants. Future studies should incorporate imaging modalities, such as intraoral radiographs, to improve diagnostic accuracy. Another important limitation of this study is that each participant was evaluated by only one orthodontist. Although all examiners underwent calibration, the inter-examiner reliability was relatively low (κ = 0.26), which may have introduced subjectivity and variability in the assessment. Further standardization efforts—such as repeated calibration sessions or the use of photographic documentation for post hoc reliability checks—may help improve consistency in future studies. Additionally, the use of photographic comparison in AC grading is inherently subjective and may vary among examiners. Although all examiners underwent calibration, future studies may benefit from the use of standardized photographic documentation and consensus-based scoring to improve reliability. Lastly, the use of chronological age was used as the sole developmental indicator in this study. However, dental age often provides a more accurate representation of individual growth stages, especially during adolescence when inter-individual variability is high. This may affect the timing and expression of malocclusion characteristics, reducing the accuracy of IOTN-based assessment. Future studies should consider incorporating dental maturity to achieve more accurate evaluations.

Building upon these findings, it is essential to consider how research outcomes can inform broader public health strategies and future technological innovations. These findings underscore the need for evidence-based criteria in school-based screening programs to address orthodontic treatment disparities and support equitable access to care, which is a growing concern in public oral health systems.

In addition, recent advances in artificial intelligence (AI) offer promising opportunities for enhancing orthodontic assessment and treatment planning. AI-based tools, including facial analysis algorithms and smartphone-based applications, have been explored for their potential to detect orthodontic anomalies, predict treatment outcomes, and support personalized care. Integrating such digital technologies into school-based or teleorthodontic screening programs could help improve access, objectivity, and diagnostic efficiency [30]. Future studies should explore the feasibility and accuracy of these AI-driven platforms in epidemiological settings.

## 5. Conclusions

This study demonstrated that crowding, increased overjet, and increased overbite were the most significant morphological indicators contributing to both orthodontic treatment need and aesthetic evaluation among junior high and high school students (aged 12–18 years). Item Response Theory analysis confirmed their high discriminatory power, while regression analysis showed their significant contribution to aesthetic perception. Furthermore, the discrepancy observed between morphological evaluation (DHC) and aesthetic evaluation (AC) suggests that aesthetic assessments alone may underestimate treatment need. These findings highlight the importance of integrating both morphological and aesthetic components in school-based orthodontic screening programs to facilitate a more accurate preliminary assessment of malocclusion severity and treatment need within the limitations of school-based screening. Further multicenter studies with larger sample sizes and consideration of potential confounding factors such as oral habits are recommended to validate these results and inform public health strategies.

## Figures and Tables

**Table 1:** (**A**) Distribution of DHC grades by school grade and gender. DHC: Dental Health Component; Grades 1 to 6 correspond to each school grade from junior high first year through senior high third year. (**B**) Distribution of Aesthetic Component (AC) grades by school grade and gender. AC: Aesthetic Component of the Index of Orthodontic Treatment Need (IOTN); Grades 1 to 6 correspond to school grades from junior high year 1 to senior high year 3.

(A)													
DHC Grade	Grade 1		Grade 2		Grade 3		Grade 4		Grade 5		Grade 6		Total
	Male	Female	Male	Female	Male	Female	Male	Female	Male	Female	Male	Female	
**1**	12	8	14	5	12	6	18	9	21	13	12	12	**142**
**2**	12	5	24	7	22	7	36	24	31	11	34	19	**232**
**3**	11	5	12	6	13	12	17	26	29	24	26	19	**200**
**4**	5	4	8	3	8	8	24	17	20	19	12	13	**141**
**5**	1	0	1	1	1	0	5	0	2	1	0	0	**12**
**Total**	**41**	**22**	**59**	**22**	**56**	**33**	**100**	**76**	**103**	**68**	**84**	**63**	**726**
**(B)**													
**AC Grade**	**Grade 1**		**Grade 2**		**Grade 3**		**Grade 4**		**Grade 5**		**Grade 6**		**Total**
	**Male**	**Female**	**Male**	**Female**	**Male**	**Female**	**Male**	**Female**	**Male**	**Female**	**Male**	**Female**	
**1**	11	5	16	7	13	7	22	23	30	16	18	17	**185**
**2**	11	6	13	4	15	8	24	17	27	17	22	15	**179**
**3**	5	2	13	4	13	9	27	13	30	16	14	10	**156**
**4**	3	5	8	1	6	4	14	14	7	7	16	11	**96**
**5**	3	0	3	1	2	1	8	4	3	3	5	1	**34**
**6**	1	1	1	0	2	1	2	2	1	5	2	0	**18**
**7**	3	1	2	3	2	1	2	2	1	2	3	1	**23**
**8**	1	1	2	1	2	2	1	1	3	2	3	5	**24**
**9**	2	1	1	0	0	0	0	0	1	0	0	1	**6**
**10**	1	0	0	1	1	0	0	0	0	0	1	1	**5**
**Total**	**41**	**22**	**59**	**22**	**56**	**33**	**100**	**76**	**103**	**68**	**84**	**62**	**726**

**Table 2:** Cross-tabulation of Dental Health Component (DHC) and Aesthetic Component (AC) grades of the Index of Orthodontic Treatment Need (IOTN), with row totals and row-wise percentage distributions. AC grades represent perceived aesthetic severity (1 = least and 10 = most), while DHC grades reflect clinical treatment need. The distribution is statistically significant by Fisher’s exact test (*p* < 0.001).

AC Grade	DHC 1	DHC 2	DHC 3	DHC 4	DHC 5	Row Total
**1**	100	62	19	4	0	**185**
**2**	24	94	49	9	3	**179**
**3**	11	39	73	33	0	**156**
**4**	3	22	37	31	3	**96**
**5**	2	5	9	16	2	**34**
**6**	0	3	4	11	0	**18**
**7**	1	5	5	10	2	**23**
**8**	1	0	2	20	1	**24**
**9**	0	2	0	3	1	**6**
**10**	0	0	2	3	0	**5**
**Column Total**	**142**	**232**	**200**	**141**	**12**	**726**

**Table 3:** Logistic models for each item of the Dental Health Component (DHC) of the Index of Orthodontic Treatment Need (IOTN), stratified by DHC grade. The table presents item-level discrimination, difficulty, and guessing parameters estimated using item response theory (IRT). “—” indicates that parameter estimates could not be computed due to insufficient data.

DHC Grade	Item Code	Discrimination	Difficulty	Guessing
**2**	a	36.15	1.1	0.22
**2**	b	−4.13	−3.3	<0.001
**2**	c	0.45	25.03	0.05
**2**	d	21.13	−0.35	<0.001
**2**	e	−0.22	−16.18	<0.001
**2**	f	25	1.45	0.16
**2**	g	1.74	2.37	0.26
**3**	a	0.89	3.63	—
**3**	b	—	7.84	—
**3**	c	—	5.11	—
**3**	d	—	0.41	—
**3**	e	—	5.81	—
**3**	f	—	3.34	—
**4**	a	48.65	1.11	0.03
**4**	b	−1.32	−5.69	<0.001
**4**	m	−1.46	−4.82	<0.001
**4**	c	−1.45	−4.82	<0.001
**4**	d	243.28	1.04	<0.001
**4**	e	2.98	2.55	<0.001
**4**	f	29.92	1.16	0.02
**4**	x	1.42	4.43	<0.001
**5**	i	3.28	2.63	—
**5**	a	−0.16	−33.46	—
**5**	m	−0.16	−42.17	—
**5**	p	−15.01	−2.57	—
**5**	s	−36.23	−3.17	—

Item codes correspond to specific malocclusion features: a: Increased overjet, b: Reverse overjet, c: Anterior or posterior crossbite, d: Crowding, e: Open bite, f: Increased overbite, g: Molar relationship, h: Cleft lip and/or palate, i: Impeded eruption of teeth, m: Reverse overjet with functional problems, *p*: Craniofacial syndrome or cleft-related defects, s: Supernumerary teeth, t: Hypodontia, x: Other anomalies (e.g., displaced teeth), I: Impacted teeth, 1: No abnormal findings. Note: Code “1” indicates cases where no abnormal morphological findings were observed; this does not correspond to DHC grade 1, which may still include minor malocclusion.

## Data Availability

All of the clinical data are available in the Supplementary Materials.

## Supplementary Materials

The following supporting information can be downloaded at: https://www.mdpi.com/article/10.3390/jcm14134802/s1, Figure S1: Flowchart of IOTN Classification and Orthodontic Treatment Need Categories. Table S1: Number of students who tested positive for each item in the Dental Health Component (DHC) of the Index of Orthodontic Treatment Need (IOTN) by DHC grade. Table S2: (A) Results of cutoff-specific logistic regression analyses for Aesthetic Component (AC) grade thresholds predicting high orthodontic treatment need (DHC grade 4 or 5). (B) Results of simple linear regression models assessing the association between individual DHC items and Aesthetic Component (AC) grade. Supplemental File S1: Individual-level data of DHC and AC assessments for all participants. Supplemental File S2: R script used for IRT analysis of DHC items.
