# Supplementary material for: Correlation Between Dental Health and Aesthetic Components of Malocclusion in Junior High and High School Students: An Epidemiological Study Using Item Response Theory
Source: J Clin Med. 2025 Jul 7;14(13):4802. doi: 10.3390/jcm14134802 (PMC12251538; doi:10.3390/jcm14134802)
Supplement: Supplementary file 1 [file jcm-14-04802-s001.zip › FigureS1 TableS1,S2.pptx]

## Slide 1
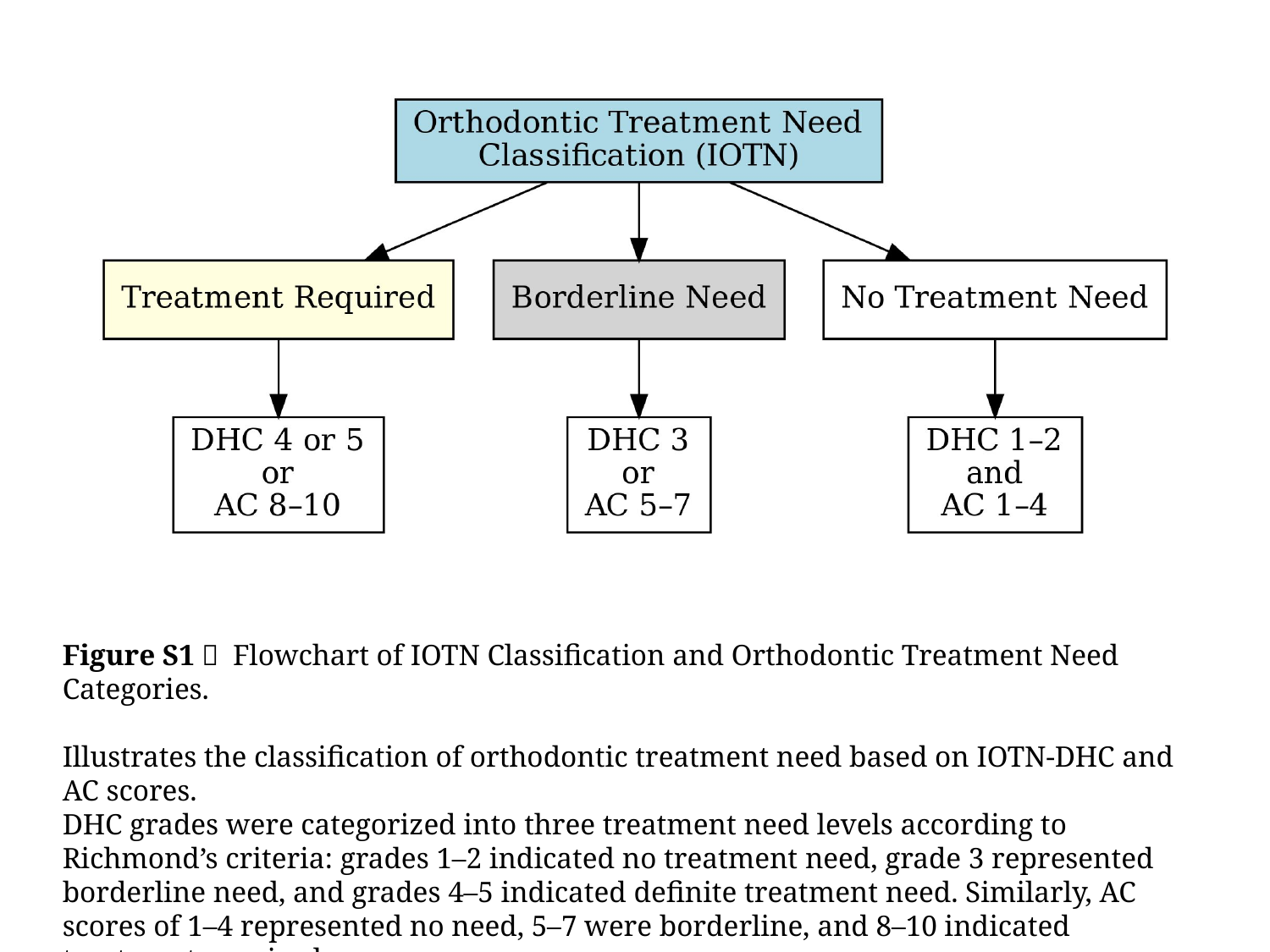

Figure S1： Flowchart of IOTN Classification and Orthodontic Treatment Need Categories.
Illustrates the classification of orthodontic treatment need based on IOTN-DHC and AC scores.
DHC grades were categorized into three treatment need levels according to Richmond’s criteria: grades 1–2 indicated no treatment need, grade 3 represented borderline need, and grades 4–5 indicated definite treatment need. Similarly, AC scores of 1–4 represented no need, 5–7 were borderline, and 8–10 indicated treatment required.

## Slide 2
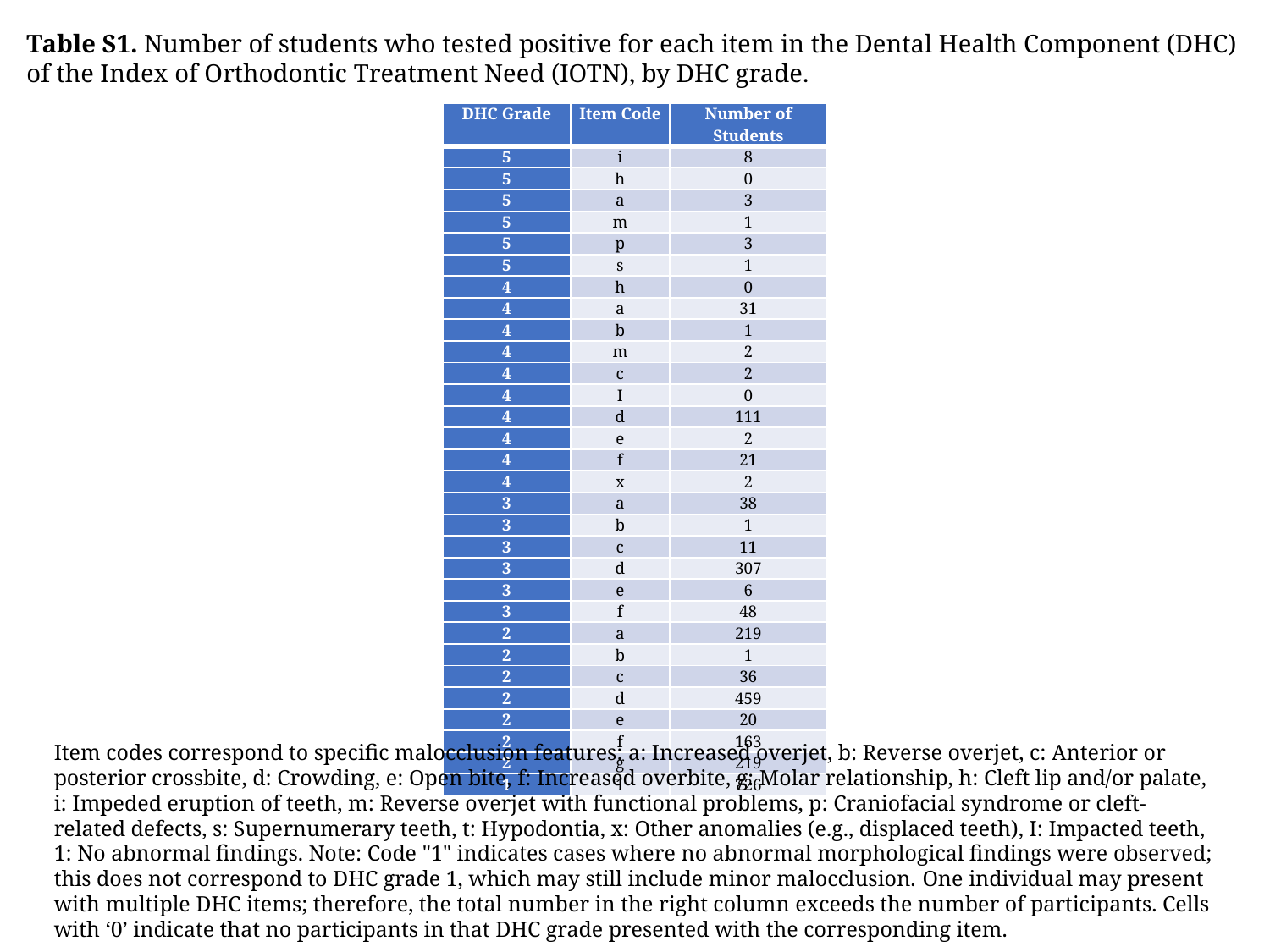

Table S1. Number of students who tested positive for each item in the Dental Health Component (DHC) of the Index of Orthodontic Treatment Need (IOTN), by DHC grade.
| DHC Grade | Item Code | Number of Students |
| --- | --- | --- |
| 5 | i | 8 |
| 5 | h | 0 |
| 5 | a | 3 |
| 5 | m | 1 |
| 5 | p | 3 |
| 5 | s | 1 |
| 4 | h | 0 |
| 4 | a | 31 |
| 4 | b | 1 |
| 4 | m | 2 |
| 4 | c | 2 |
| 4 | I | 0 |
| 4 | d | 111 |
| 4 | e | 2 |
| 4 | f | 21 |
| 4 | x | 2 |
| 3 | a | 38 |
| 3 | b | 1 |
| 3 | c | 11 |
| 3 | d | 307 |
| 3 | e | 6 |
| 3 | f | 48 |
| 2 | a | 219 |
| 2 | b | 1 |
| 2 | c | 36 |
| 2 | d | 459 |
| 2 | e | 20 |
| 2 | f | 163 |
| 2 | g | 219 |
| 1 | 1 | 726 |
Item codes correspond to specific malocclusion features: a: Increased overjet, b: Reverse overjet, c: Anterior or posterior crossbite, d: Crowding, e: Open bite, f: Increased overbite, g: Molar relationship, h: Cleft lip and/or palate, i: Impeded eruption of teeth, m: Reverse overjet with functional problems, p: Craniofacial syndrome or cleft-related defects, s: Supernumerary teeth, t: Hypodontia, x: Other anomalies (e.g., displaced teeth), I: Impacted teeth, 1: No abnormal findings. Note: Code "1" indicates cases where no abnormal morphological findings were observed; this does not correspond to DHC grade 1, which may still include minor malocclusion. One individual may present with multiple DHC items; therefore, the total number in the right column exceeds the number of participants. Cells with ‘0’ indicate that no participants in that DHC grade presented with the corresponding item.

## Slide 3
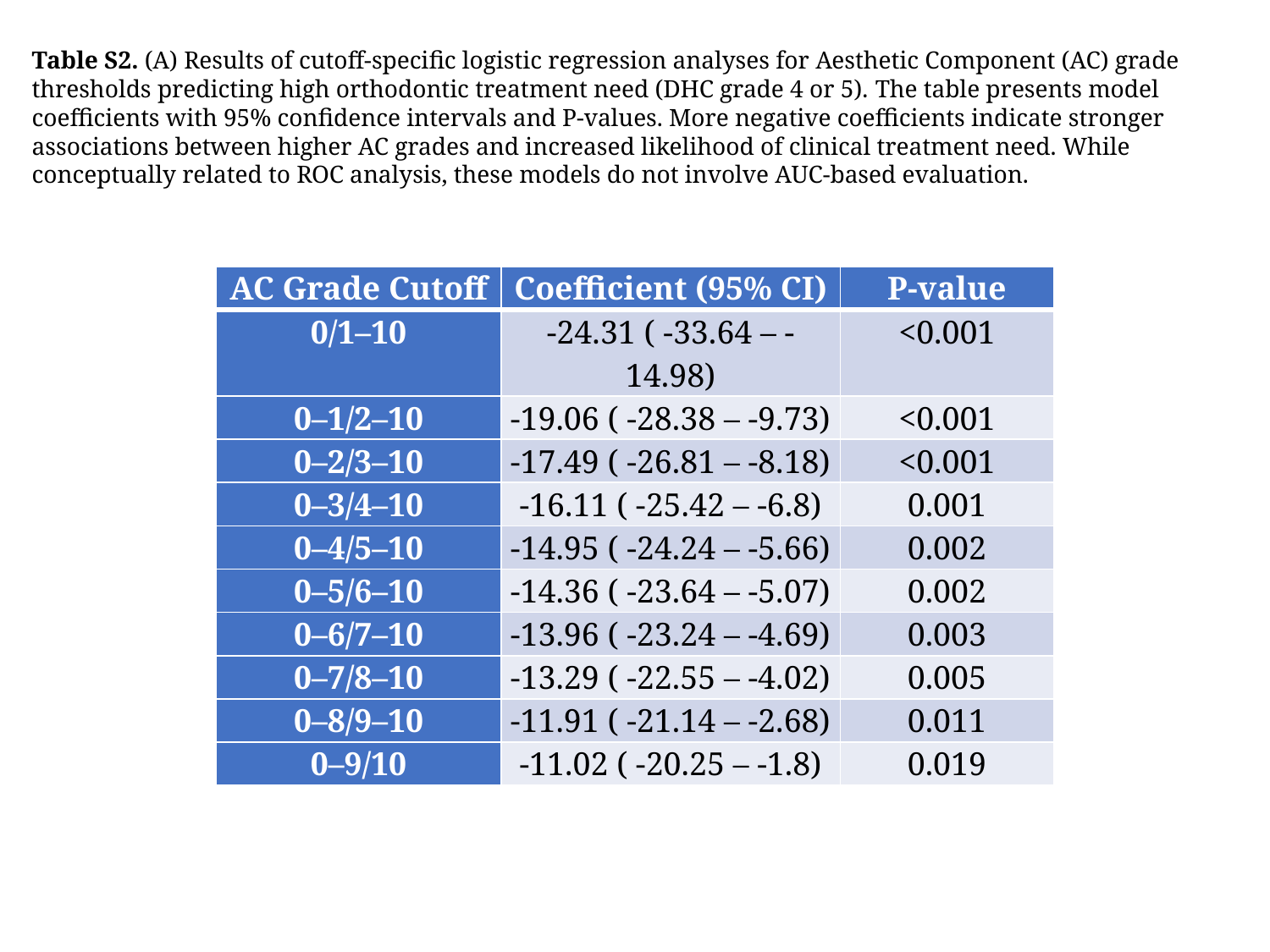

Table S2. (A) Results of cutoff-specific logistic regression analyses for Aesthetic Component (AC) grade thresholds predicting high orthodontic treatment need (DHC grade 4 or 5). The table presents model coefficients with 95% confidence intervals and P-values. More negative coefficients indicate stronger associations between higher AC grades and increased likelihood of clinical treatment need. While conceptually related to ROC analysis, these models do not involve AUC-based evaluation.
| AC Grade Cutoff | Coefficient (95% CI) | P-value |
| --- | --- | --- |
| 0/1–10 | -24.31 ( -33.64 – -14.98) | <0.001 |
| 0–1/2–10 | -19.06 ( -28.38 – -9.73) | <0.001 |
| 0–2/3–10 | -17.49 ( -26.81 – -8.18) | <0.001 |
| 0–3/4–10 | -16.11 ( -25.42 – -6.8) | 0.001 |
| 0–4/5–10 | -14.95 ( -24.24 – -5.66) | 0.002 |
| 0–5/6–10 | -14.36 ( -23.64 – -5.07) | 0.002 |
| 0–6/7–10 | -13.96 ( -23.24 – -4.69) | 0.003 |
| 0–7/8–10 | -13.29 ( -22.55 – -4.02) | 0.005 |
| 0–8/9–10 | -11.91 ( -21.14 – -2.68) | 0.011 |
| 0–9/10 | -11.02 ( -20.25 – -1.8) | 0.019 |

## Slide 4
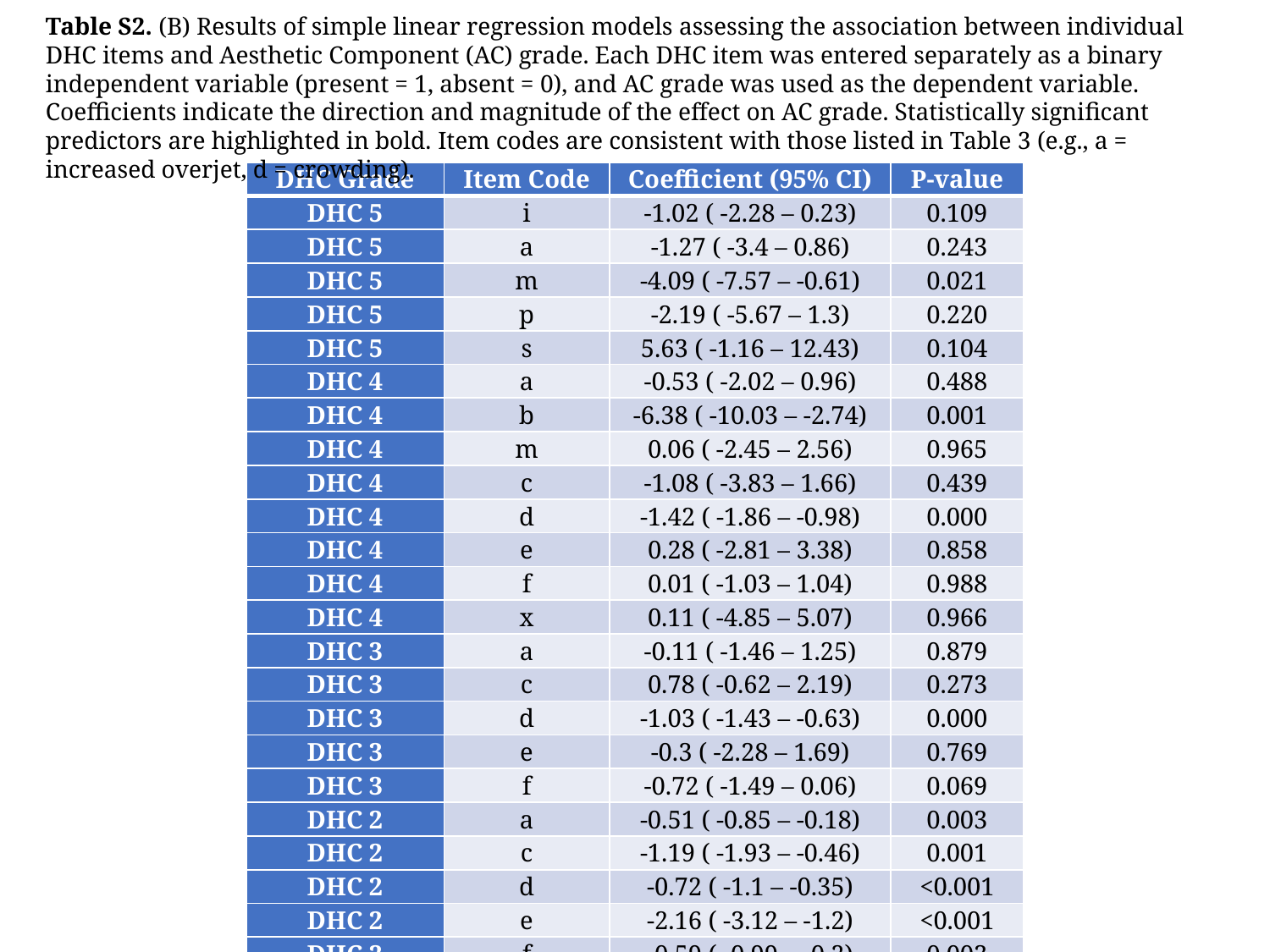

Table S2. (B) Results of simple linear regression models assessing the association between individual DHC items and Aesthetic Component (AC) grade. Each DHC item was entered separately as a binary independent variable (present = 1, absent = 0), and AC grade was used as the dependent variable. Coefficients indicate the direction and magnitude of the effect on AC grade. Statistically significant predictors are highlighted in bold. Item codes are consistent with those listed in Table 3 (e.g., a = increased overjet, d = crowding).
| DHC Grade | Item Code | Coefficient (95% CI) | P-value |
| --- | --- | --- | --- |
| DHC 5 | i | -1.02 ( -2.28 – 0.23) | 0.109 |
| DHC 5 | a | -1.27 ( -3.4 – 0.86) | 0.243 |
| DHC 5 | m | -4.09 ( -7.57 – -0.61) | 0.021 |
| DHC 5 | p | -2.19 ( -5.67 – 1.3) | 0.220 |
| DHC 5 | s | 5.63 ( -1.16 – 12.43) | 0.104 |
| DHC 4 | a | -0.53 ( -2.02 – 0.96) | 0.488 |
| DHC 4 | b | -6.38 ( -10.03 – -2.74) | 0.001 |
| DHC 4 | m | 0.06 ( -2.45 – 2.56) | 0.965 |
| DHC 4 | c | -1.08 ( -3.83 – 1.66) | 0.439 |
| DHC 4 | d | -1.42 ( -1.86 – -0.98) | 0.000 |
| DHC 4 | e | 0.28 ( -2.81 – 3.38) | 0.858 |
| DHC 4 | f | 0.01 ( -1.03 – 1.04) | 0.988 |
| DHC 4 | x | 0.11 ( -4.85 – 5.07) | 0.966 |
| DHC 3 | a | -0.11 ( -1.46 – 1.25) | 0.879 |
| DHC 3 | c | 0.78 ( -0.62 – 2.19) | 0.273 |
| DHC 3 | d | -1.03 ( -1.43 – -0.63) | 0.000 |
| DHC 3 | e | -0.3 ( -2.28 – 1.69) | 0.769 |
| DHC 3 | f | -0.72 ( -1.49 – 0.06) | 0.069 |
| DHC 2 | a | -0.51 ( -0.85 – -0.18) | 0.003 |
| DHC 2 | c | -1.19 ( -1.93 – -0.46) | 0.001 |
| DHC 2 | d | -0.72 ( -1.1 – -0.35) | <0.001 |
| DHC 2 | e | -2.16 ( -3.12 – -1.2) | <0.001 |
| DHC 2 | f | -0.59 ( -0.99 – -0.2) | 0.003 |
| DHC 2 | g | -0.93 ( -1.23 – -0.62) | <0.001 |
| DHC 1 | — | -2.32 ( -8.09 – 3.45) | 0.431 |
