# Supplementary material for: Correlation Between Dental Health and Aesthetic Components of Malocclusion in Junior High and High School Students: An Epidemiological Study Using Item Response Theory
Source: J Clin Med. 2025 Jul 7;14(13):4802. doi: 10.3390/jcm14134802 (PMC12251538; doi:10.3390/jcm14134802)
Supplement: Supplementary file 1 [file jcm-14-04802-s001.zip › Supplemental File S2.pdf]

```
library(ltm)
library(irtoys)
DATA2<-read.csv("2.csv")
model1<-tpm(DATA2)
item.fit(model1)
F1<-factor.scores(model1, resp.pattern= DATA2)
F1
DATA2<-read.csv("2.csv")
MDATA2<-as.matrix(DATA2)
RESULTPL2 <-est　(resp=MDATA2, model="3PL", engine="ltm")
RESULTPL2
value2<-irf(RESULTPL2)
plot(value2,label=TRUE,co=NA)
valuesIF2<-iif(RESULTPL2)
plot(valuesIF2, label=TRUE, co=NA)
value2
valuesIF2
```

```
DATA3<-read.csv("3.csv")
model3<-tpm(DATA3)
item.fit(model3)
F3<-factor.scores(model3, resp.pattern= DATA3)
F3
DATA3<-read.csv("3.csv")
MDATA3<-as.matrix(DATA3)
RESULTPL3 <-est (resp=MDATA3, model="1PL", engine="ltm")
RESULTPL3
value3<-irf(RESULTPL3)
plot(value3,label=TRUE,co=NA)
valuesIF3<-iif(RESULTPL3)
plot(valuesIF3, label=TRUE, co=NA)
value3
valuesIF3
```

```
DATA4<-read.csv("4.csv")
model4<-tpm(DATA4)
item.fit(model4)
F4<-factor.scores(model4, resp.pattern= DATA4)
F4
DATA4<-read.csv("4.csv")
MDATA4<-as.matrix(DATA4)
RESULTPL4 <-est (resp=MDATA4, model="3PL", engine="ltm")
RESULTPL4
value4<-irf(RESULTPL4)
plot(value4,label=TRUE,co=NA)
valuesIF4<-iif(RESULTPL4)
plot(valuesIF4, label=TRUE, co=NA)
value4
valuesIF4
```

```
DATA5<-read.csv("5.csv")
model5<-tpm(DATA5)
item.fit(model5)
F5<-factor.scores(model5, resp.pattern= DATA5)
F5
DATA5<-read.csv("5.csv")
MDATA5<-as.matrix(DATA5)
RESULTPL5 <-est (resp=MDATA5, model="2PL", engine="ltm")
RESULTPL5
value5<-irf(RESULTPL5)
plot(value5,label=TRUE,co=NA)
valuesIF5<-iif(RESULTPL5)
plot(valuesIF5, label=TRUE, co=NA)
value5
valuesIF5
```
